# The Effectiveness of a Community-Based Mentoring Program for Children Aged 5–11 Years: Results from a Randomized Controlled Trial

**DOI:** 10.1007/s11121-020-01132-4

**Published:** 2020-07-28

**Authors:** Nick Axford, Gretchen Bjornstad, Justin Matthews, Laura Whybra, Vashti Berry, Obioha C. Ukoumunne, Tim Hobbs, Zoe Wrigley, Lucy Brook, Rod Taylor, Tim Eames, Angeliki Kallitsoglou, Sarah Blower, Georgina Warner

**Affiliations:** 1 NIHR ARC South West Peninsula (PenARC), University of Plymouth, Plymouth, UK; 2 University of Exeter Medical School, Exeter, UK; 3 PenARC, University of Exeter, Exeter, UK; 4 Dartington Service Design Lab, Dartington, UK; 5 Cardiff University, Cardiff, UK; 6 Devon Partnership NHS Trust, Exeter, UK; 7 University of Roehampton, London, UK; 8 NIHR ARC Yorkshire and Humber, University of York, York, UK; 9 Uppsala University, Uppsala, Sweden

**Keywords:** Mentoring, Behavioral and emotional problems, Randomized controlled trial, Effectiveness research, Early intervention

## Abstract

The study, a two-arm, randomized controlled, parallel group, superiority trial, aimed to evaluate the implementation and effectiveness of a 12-month one-to-one volunteer mentoring program designed to improve behavioral and emotional outcomes in children aged 5 to 11 years who have teacher- and parent/carer-reported behavioral difficulties. Participants were 246 children (123 intervention, 123 control; mean age 8.4 years; 87% boys) in five sites in London, UK, scoring in the “abnormal” range on the teacher-rated Strengths and Difficulties Questionnaire (SDQ) Total Difficulties measure and in the “borderline” or abnormal range on the parent-rated SDQ Total Difficulties measure. Randomization on a 1:1 ratio took place using a computer-generated sequence and stratifying by site. Data collectors and statisticians were blind to participant allocation status. Outcome measures focused on parent- and teacher-rated child behavior and emotions, and child-rated self-perception and hope. Intention-to-treat analysis on all 246 randomized participants (using imputed data where necessary) showed that at post-intervention (16 months after randomization), there were no statistically significant effects on the primary outcome—parent-rated SDQ Total Difficulties (adjusted standardized mean difference = − 0.12; 95% CI: −0.38 to 0.13; *p* = 0.33)—or any secondary outcomes. Results from complier average causal effect (CACE) analysis using the primary outcome indicated the intervention was not effective for children who received the recommended duration of mentoring. Exploratory analyses found no sub-group effects on the primary outcome. The article concludes that the mentoring program had no effect on children’s behavior or emotional well-being, and that program content needs revising to satisfactorily address key risk and protective factors.

**Electronic supplementary material:**

The online version of this article (10.1007/s11121-020-01132-4) contains supplementary material, which is available to authorized users.

## Introduction

Serious antisocial behavior in adolescence and adulthood can be predicted by early signs of behavioral and emotional difficulties in childhood (Farrington and Welsh 2007). Individual-level risk factors for antisocial behavior often express themselves as impulsiveness, difficulties in relating well to peers, poor problem-solving skills, and an inability to regulate conduct and emotions (Rutter et al. 1998; Moffitt and Scott 2008). Left untreated, childhood behavioral and emotional difficulties, which affect approximately 10% of children aged 5–15 years in Britain (Green et al. 2005), elevate children’s risk for poor outcomes across multiple domains, including academic achievement, health, social relationships, and offending (Nagin and Tremblay 1999; Roza et al. 2003; Patel et al. 2007; Breslau et al. 2008; Bailey et al. 2009; Calkins and Keane 2009; Fletcher 2010). For this reason, it is important to prevent such difficulties and thereby avert later antisocial and criminal behavior.

Mentoring programs offer one approach to preventing childhood behavioral and emotional difficulties, the primary outcome in the trial reported here. They involve forging a strong personal connection between a child and positive adult role model who enables the child to take part in positive activities and commit to socially appropriate goals. This relationship is theorized to improve developmental outcomes (e.g., behavior, emotional well-being, academic attainment) by catalyzing developmental processes in children’s social-emotional, cognitive, and identity development, which in turn enable them to interact better with parents and peers (Rhodes and Dubois 2008). For example, *identity* development is promoted by the mentor encouraging a more positive future orientation, displaying qualities that youth might wish to emulate and exposing mentees to new contexts and resources, thereby expanding their range of possible selves; *cognitive* development, including self-regulation, is encouraged through shared activities and meaningful conversations with more sophisticated thinkers; and *social-emotional* development is furthered by the mentor facilitating more positive connections with others (peers and adults) and providing a context in which to interpret and manage interpersonal difficulties.

In recent years, mentoring interventions have become an increasingly popular low-cost strategy for early intervention with at-risk youth but questions remain about the extent to which they are effective (Raposa et al. 2019). The most recent comprehensive meta-analytic reviews prior to the present study commencing showed effect sizes across outcomes ranging from 0.18 to 0.21 (DuBois et al. 2002, 2011). Although considered small according to conventional guidelines, these hide variability across studies, with some effect sizes in the medium and large range. Moderator analyses identified factors associated with stronger effects: matching the young person with the correct mentor based on shared interests; including structured activities, particularly if driven by the young person’s needs and interests; targeting youth who demonstrate behavioral difficulties; including a parent support and involvement element; a duration of 12 months or longer; holding mentor/youth meetings at least once a week; and providing mentor training and support (DuBois et al. 2011).

Chance UK, a non-governmental organization working in London since 1995, developed and delivers a 12-month one-to-one mentoring program for children aged 5 to 11 years who display challenging behavior and emotional problems at school and home. It aims to prevent future antisocial and criminal behavior (distal outcomes) by reducing early behavioral and emotional difficulties (proximal outcomes). Trained and supervised mentors build strong relationships with children, serve as positive role models, and provide access to new opportunities and networks. They seek to help participants develop the following: (i) improved self-esteem (by identifying strengths, for instance in creative, sporting, or academic arenas, and providing positive feedback on prosocial behavior); (ii) greater self-efficacy (by encouraging participation in activities and helping children to see how their efforts yield positive effects); (iii) better social and relationship skills, including regulation of conduct and emotions (by modeling such behavior and role-playing challenging scenarios); and (iv) higher aspirations (by exposing children to different experiences and discussing their potential and preferred futures). Collectively, these factors reflect the three developmental emphases cited above (identity, cognitive, social-emotional) and contribute to positive child behavior and emotional well-being, the primary program focus (e.g., Catalano and Hawkins 1996; Donnellan et al. 2005; Sowislo and Orth 2013; Wigelsworth et al. 2017). The program is underpinned by a solution-focused approach (Ratner et al. 2012; Bond et al. 2013). This encourages a positive future orientation by identifying goals and steps for getting there (identity development), helps the child to reflect on their actions and identify effective behaviors they have used to cope with difficult situations (cognitive development), and encourages the child to identify their strengths and thereby build positive self-esteem (social-emotional development).

The intervention’s core design embodies the features of more effective mentoring programs cited earlier. It targets children with identified behavioral and emotional difficulties; volunteer mentors are trained and supervised to deliver a tailored program of structured activities; a thorough matching process operates, based on the mentor’s personality and characteristics; sessions take place weekly for 12 months; and parents are offered support. It also works with younger children than is common in studies of mentoring programs to date, in other words, when children’s behavior may be more malleable (Loeber 1991; Bywater 2012). For these reasons, it was reasonable to expect that Chance UK’s program would have a larger effect size than the mean effect found in the DuBois et al. (2011) meta-analysis.

Prior to this trial, Chance UK’s mentoring program had been evaluated in a pre-post study (Smith and Howard 2008). The mean parent-rated Strengths and Difficulties Questionnaire (SDQ) Total Difficulties score fell from 19.25 out of 40 (for the 99 children entering the program) to 14.82 (based on data for 92 children)^1^ after a year of mentoring (*p* < 0.001), while the mean teacher-rated SDQ Total Difficulties score decreased from 23.41 to 16.48 (*p* < 0.001). A definitive trial was justified given ongoing questions about youth mentoring effectiveness and the fact that although the specific intervention was well established, there was no prior trial of youth mentoring in the UK.

The objectives of this trial were as follows: (1) to estimate the effect that offering the Chance UK mentoring program has on children’s behavior and socio-emotional well-being (the primary outcome) in comparison to similar children who are not offered the program; (2) to estimate the effect that the program has on children’s self-esteem and self-efficacy, both of which are hypothesized mediators of intervention effect; and (3) to describe the extent to which the program is implemented with fidelity to the program design. It was hypothesized that, compared with children who are not offered mentoring (control arm), children who are offered mentoring (intervention arm) will, post intervention, have fewer emotional and behavioral difficulties (reported by parent/carers) and higher self-esteem and self-efficacy (child self-report).

## Method

### Design

The study was an independent two-arm, randomized controlled, parallel group, superiority trial designed to evaluate the effectiveness of Chance UK’s mentoring program in improving behavioral and emotional outcomes in primary school children who have teacher- and parent/carer-reported behavioral difficulties. The intervention arm was offered the mentoring program; both trial arms had access to services as usual. Assessments took place at pre-intervention (baseline: between July 2014 and March 2016), mid-way through the mentoring year (9 months after randomization, midpoint: April 2015 to December 2016) and post-mentoring program (16 months after randomization, endpoint: November 2015 to July 2017). The methods are elaborated on in the published protocol (Whybra et al. 2018).

### Setting

Chance UK delivered the intervention in community settings in five London boroughs: Enfield, Hackney, Islington, Lambeth, and Waltham Forest. All have a high proportion of children from minority ethnic groups, relatively high rates of child poverty, and a large proportion of rented accommodation (Tables S1 and S2). Assessments for the RCT took place by phone and in the home and school (online for teachers).

### Participants and Procedure

Children were eligible to participate in the study if they were aged between 5 and 10 years at referral (meaning the child would be aged 5 to 11 during mentoring); lived or attended school in one of the five boroughs; and scored ≥ 16 on teacher-reported SDQ (TSDQ) Total Difficulties (“abnormal” range) and ≥ 14 on parent/carer-reported SDQ (PSDQ) Total Difficulties (“borderline” (14–16) or abnormal (17–40) ranges). Children were ineligible if any of the following applied: there was a diagnosis of autism or a developmental delay that would prevent them from engaging in the program and the study; information supplied by the child’s school to Chance UK at referral indicated a risk of violence towards Chance UK staff or the research team by the child or parent/carer; or a child’s sibling was enrolled in the study.

Recruitment took place between May 2014 and February 2016. Children were referred to the trial by a member of school staff who knew the child well (e.g., a class teacher or Special Educational Needs Coordinator (SENCO)) and who had concerns about the child’s behavior. In order to ensure that suitable candidates were referred, school staff were given printed materials about the program, directed to the Chance UK website, encouraged to refer children with challenging behavior and/or who were excluded or at risk of exclusion, and advised that the program is not for children with moderate or severe learning difficulties. Chance UK was responsible for sourcing referrals and screened each completed referral form, which contains the TSDQ, to check eligibility for the study. Each suitable referral was passed to the trial coordinator who contacted the main parent/carer by telephone to explain more about the program and study and to conduct further eligibility checks, including the baseline PSDQ. Where parents/carers were interested and the child met the initial eligibility criteria, an independent data collector visited the family home to obtain written informed consent and collect additional baseline measures prior to randomization. Strategies to minimize attrition from the trial were described in the protocol paper.

### Sample Size

The sample size was calculated in STATA based on a comparison of the means of the primary outcome between the intervention and control arms. Two hundred forty-six eligible children needed to be recruited to detect an effect size of 0.4 with 80% power at the 5% level of significance, allowing for a study drop-out of up to 20% (an effect size of 0.4 requires a minimum sample size of 99 participants per arm).

### Randomization

Participants were randomly allocated using a 1:1 ratio to intervention and control arms using a computer-generated randomization sequence, stratified by site (Enfield, Hackney, Islington, Lambeth, and Waltham Forest). In each location, the first 25% of children were allocated by simple randomization and thereafter minimization was used to reduce imbalance between the program and control groups in terms of age (< 9 versus ≥ 9 years) and gender (male versus female). Randomization took place after baseline data collection and employed a dynamic approach, meaning that each participant could be randomized as soon as they had completed baseline assessments. The allocation sequence was concealed using an online central randomization service set up and maintained by the Exeter Clinical Trials Network. The principal investigator, trial manager, data collectors, and statisticians were blind to participant allocation status.

### Control Arm

Children assigned to the control arm were permitted to receive services as usual, because the aim of the trial is to determine whether the mentoring program provides added value. Prior to the trial, Chance UK stated that the services on offer would vary between boroughs but would likely include clubs, scouts, after school activities, CAMHS (Child and Adolescent Mental Health Services), and youth projects. Other services were considered unlikely to resemble the Chance UK intervention, as early investigation suggested that few, if any, mentoring programs were available in relevant boroughs. In addition, referrers were signposted to a standard universal children’s services directory available to each London borough.

### Intervention Arm

Children in the intervention arm were offered the Chance UK mentoring program. This comprises weekly one-to-one mentoring sessions, each intended to last 2 to 4 h, over 12 months. A matching exercise overseen by Chance UK pairs each child with a trained mentor based on several factors, including the mentor’s personality, shared interests (with the child), parent preferences, and mentor availability. Matches are usually successful, meaning that they do not break down; those that break down are usually owing to practical issues, such as changes in the life circumstances of the mentor (e.g., bereavement, change of job) or the family (e.g., moving outside catchment area, entering care). There were 123 different mentors in the trial, one for each participant offered the intervention. Mentors develop a program of interactive activities tailored to their child’s interests and needs. The sessions aim to help children to (i) progress to their identified “preferred future” by working towards specified personal goals (e.g., regarding family relationships, activities they enjoy, education), (ii) recognize and build their strengths (e.g., trying hard, exhibiting prosocial behavior), and (iii) consider and try out more effective responses to difficulties (e.g., role-playing prosocial ways of dealing with frustration or anger), all while giving them access to networks and opportunities that would otherwise be unavailable to them.

The mentor uses solution-focused techniques to help improve child behavior without exploring the behavior’s root cause: (i) problem-free talk (e.g., amplifying positives, asking questions, reframing issues); (ii) identifying and encouraging the child’s strengths (e.g., challenging negative statements they make about themselves based on previous experiences); (iii) giving positive and specific feedback about what a child has done well in a particular situation (e.g., if they tried hard); and (iv) imagining a preferred future by helping a child to identify where they are on a particular issue, where they want to be, and how that can be achieved (e.g., identifying what they can influence and working together on that issue).

The first 3 months of mentoring focus on building a trusting relationship between child and mentor and identifying the child’s difficulties and strengths. The mentor, child, main parent/carer, and Chance UK then meet to agree at least one behavioral goal, one educational or social skills goal, and one fun goal. There are also often implicit goals known to the mentor and project manager, such as helping the child to deal with anger. The remainder of the mentoring year focuses on achieving these goals and building the child’s strengths. Each child may also choose to attend one or more group mentoring sessions with other children and mentors. After 9 months, the mentor and the child start preparing for a positive end to the mentoring relationship. A graduation ceremony attended by family and friends marks the end of the year and celebrates the child’s successes and goals achieved.

In an optional part of the intervention, taken up by those who are interested, Chance UK works with the child’s parent/carer(s). This applies the solution-focused approach and may involve practical assistance with family management, assisting with personal development such as preparing a CV, or signposting and introduction to relevant services. Support can be offered through one-to-one sessions, family group sessions, or group workshops. The parent/carer service can take place throughout the mentoring program.

Mentors complete a 3-day training delivered by Chance UK staff and covering the following: intervention aims and objectives, program structure and logic model, the solution-focused approach, safeguarding, and reporting requirements. Training is delivered in a group setting and involves extensive role play, individual feedback, and discussion. Trainees are also given homework tasks (e.g., to prepare a presentation exploring the perspectives on mentoring of parents or referrers).

### Outcome Measures

Outcome measures were selected to reflect key elements of the program theory of change. The parent-reported SDQ (Goodman 1997) Total Difficulties score is the primary outcome; all other outcomes are secondary. All measures have been shown in previous studies to have good internal validity and reliability (Whybra et al. 2018), and internal consistency in the current sample is at least acceptable (Cronbach’s alpha ≥ 0.7) for all measures at all time points except two (Table S3).

The SDQ is a widely used 25-item questionnaire for measuring children’s behavioral and emotional difficulties (Goodman 1997). This study included the Parent-report (PSDQ) and the Teacher-report (TSDQ) versions for children aged 4–17 years. Each contains five subscales of five items, assessing conduct problems, emotional problems, hyperactivity, peer problems, and prosocial behavior respectively. The first four of these are summed to provide a Total Difficulties score (primary outcome) with a range of 0 to 40, where higher scores indicate greater difficulties. This score can be categorized into “Normal” (0–13 PSDQ, 0–11 TSDQ), Borderline (14–16 PSDQ, 12–15 TSDQ), and Abnormal (17–40 PSDQ, 16–40 TSDQ). The SDQ also includes a brief Impact Supplement, focused on the impact of behavioral and/or socio-emotional difficulties on the child, their everyday life, and the people around them. The PSDQ Impact Score ranges from 0 to 10, and the TSDQ Impact Score ranges from 0 to 6, with a higher score indicating a greater impact.

The Eyberg Child Behavior Inventory (ECBI) (Eyberg and Ross 1978) is a 36-item parent/carer-rated measure of behavior problems exhibited by children aged 2 to 16 years, with two scales: an Intensity Scale (scoring range 36 to 252, indicating low to high frequency of common behavior problems) and a Problem Scale (scoring range 0 to 36, indicating low to high extent to which behaviors are deemed problematic). The ECBI is more sensitive than the SDQ.

The Self-Perception Profile for Children (SPPC) (Harter 1982, 2012) is a 36-item self-report measure comprising six six-item scales, four of which are used here, all assessed at endpoint: global self-worth, scholastic competence, social competence, and behavioral conduct. Each scale score is obtained by calculating the mean response score for the relevant items, with scores ranging from 1 (lower self-perceived competence) to 4 (higher self-perceived competence). This scale was used to measure children’s self-esteem (for those aged 8 years and above at baseline).

The Children’s Hope Scale (CHS) (Snyder et al. 1997) is a six-item self-report measure with two three-item subscales, assessing whether children feel able to initiate and move towards goals (agency subscale) and create a plan to work towards their goals (pathway subscale). The overall score is calculated by adding the responses to the six items, with scores ranging from 6 to 36 (higher scores are better). This scale was used to measure children’s self-efficacy (for those aged 8 years and above at baseline).

### Other Measures

The Family Demographics Questionnaire (FDQ) was used at baseline to gather information about the child and their family. It is adapted from one used in a parenting intervention trial (Hutchings et al. 2007) and includes date of birth, age, gender, ethnicity, SEN status, education, household members, relationship quality, family health, and financial situation. Chance UK recorded mentors’ gender, age, ethnicity, and employment status.

The Family Service Use Questionnaire (FSUQ) was administered to the parent/carer at midpoint and endpoint to record families’ receipt of targeted school services and additional services, detailing the typical length and number of contacts. It is a modified version of the widely used Client Service Receipt Inventory (CSRI) (Chisholm et al. 2000).

The Beck Depression Inventory II (BDI-II) Short Form (Beck and Beck 1972) was used to measure maternal cognitive-affective symptoms at baseline, midpoint, and endpoint. Thirteen items cover areas such as sadness, loss of pleasure, self-dislike, and crying, with scores ranging from 0 to 39 (higher scores indicate more severe depression). There is some evidence that maternal depression is associated with a tendency for mothers to over-report child behavior problems (Fergusson et al. 1993; Najman et al. 2000), so the score was adjusted for in the comparisons between trial arms.

### Implementation Fidelity

Fidelity data were collected from three sources: the Program Manager (PM) (following each monthly supervision session), the child (at 3 and 9 months), and Parent Program Managers (PPM) (for each parent and family session). Four dimensions of fidelity were measured (see also Table S4): *dose*—the number and length of mentoring sessions (PM) and amount of additional support for children, parents and families (PPM); *adherence*—the mentor’s use of solution-focused techniques (7 items (e.g., “problem-free talk”), yes/no responses, range 0 (low) to 7 (high)) (PM); *quality*—rating of quality of the mentoring provided (10 items (e.g., “mentor engages the child in interactive tasks with a purpose”), 3-point scale (“good,” “acceptable,” “improvement needed”), range 10 (low) to 30 (high)) (PM); and *engagement*—child-completed Mentor Youth Alliance Scale (MYAS; Zand et al. 2009), which measures the child’s feelings of compatibility with the mentor and satisfaction with different aspects of the mentoring relationship (10 items (e.g., “My mentor cares about me”), 4-point response scale (from “very false” to “very true”), range 10 (low) to 40 (high)). The MYAS has good validity and reliability, including a Cronbach’s alpha of 0.85 (Zand et al. 2009). Additional aspects of implementation measured were time taken to match children with mentors, extent of breakdown in matches, and mentor rating of the support received from their program manager.

### Data Analysis

The comparison of outcomes was conducted according to the principle of intention-to-treat and included all 246 participants, analyzed according to the trial arm to which they were randomized. Trial arms were compared in crude (unadjusted) analyses. Linear regression (for continuous outcomes) and logistic regression (for binary outcomes) were used to adjust these comparisons for the baseline score of the outcome in question, variables used to balance the randomization (site, age group, gender), ethnicity, SEN, SES, and baseline BDI-II score. The adjusted analysis is considered primary. In exploratory analyses, tests of interaction were used to examine whether the program effect differs across various socio-demographic categories and the baseline level of total PSDQ total difficulties. The findings are based on analyses of 20 multiply imputed datasets to handle missing data. All outcome analyses were carried out using R software 3.5.0 (R Core Team 2018).

Fidelity was summarized using descriptive statistics, focusing on the different dimensions measured (adherence, dose, quality, engagement). The protocol planned for a complier average causal effect analysis (CACE) (Hewitt et al. 2006; Dunn and Bentall 2007; Stuart et al. 2008) to quantify the intervention effect on the primary outcome (endpoint PSDQ Total Difficulties) on children who attend 11 or more months of mentoring before endpoint (the recommended amount). The CACE analysis compares “compliers” in the intervention arm (those who “comply” with the intervention offered, in this case attending mentoring sessions fully) with a comparable group in the control arm (those who would have complied had they—counterfactually—been offered the intervention). Exploratory and unplanned CACE analyses examined other fidelity variables. These were put in binary form if required, with thresholds chosen independently and prior to analysis. An individual was treated as complying (or not) if they crossed the associated threshold (or not).

## Results

### Baseline Characteristics

**Fig. 1:**
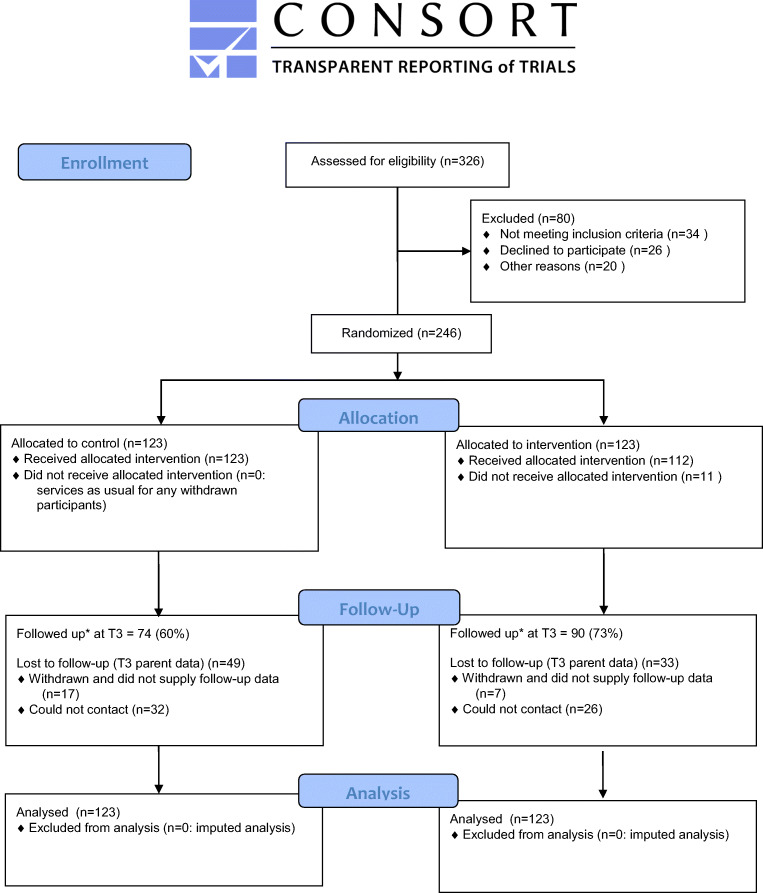
CONSORT 2010 flow diagram; *for primary outcome

The CONSORT diagram (Fig. 1) depicts the flow of referral, recruitment, and retention in the trial. The randomized sample comprised 246 children (intervention *N* = 123, control *N* = 123). Most of the sample were boys (87.4%), and the mean age at baseline was 8.4 years (standard deviation = 1.2). One quarter of participants came from households that were struggling financially, defined as finding it “very” or “extremely” difficult to live on current household income.

At baseline, intervention and control arms were similar for many characteristics, including age, gender, parent marital status, and SES (Table 1). There were some differences; for example, in the intervention arm there was a higher proportion of minority ethnic group children (66.7% intervention vs. 56.6% control) and a higher proportion of children from families in the lowest income category (34.2% vs. 27.9%). Intervention and control arms were very similar at baseline on all outcome measures (Table S5). Attrition (participants withdrawn or unable to contact) by the endpoint was 49 for the control arm (40%) and 33 for the intervention arm (27%) (Fig. 1). After attrition, there was good equivalence between arms on all baseline outcome measures but an imbalance on some demographic characteristics (Tables 1 and S4).

**Table 1:** Baseline demographic characteristics of all participants in the trial (by arm), those lost to follow-up (withdrawn or unable to contact), and those remaining in the trial to the end. Values are percentages (number) unless stated otherwise

Baseline variable	All participants		Participants lost to follow-up		Remaining participants	
	Intervention (N=102 to 123)	Control (N=109 to 123)	Intervention (N=26 to 33)	Control (N=43 to 49)	Intervention (N=76 to 90)	Control (N=66 to 74)
*Child age*						
< 9 years	50.4 (62)	48.8 (60)	54.5 (18)	38.8 (19)	48.9 (44)	55.4 (18)
≥ 9 years	49.6 (61)	51.2 (63)	45.5 (15)	61.2 (30)	51.1 (46)	44.6 (33)
*Gender*						
Male	87.0 (107)	87.8 (108)	81.8 (27)	87.8 (43)	88.9 (80)	87.8 (65)
Female	13.0 (16)	12.2 (15)	18.2 (6)	12.2 (6)	11.1 (10)	12.2 (9)
*Ethnicity*						
White	33.3 (40)	43.4 (53)	41.9 (13)	43.8 (21)	30.3 (27)	43.2 (32)
Asian/Asian British	5.7 (7)	2.5 (3)	3.2 (1)	0.0 (0)	6.7 (6)	4.1 (3)
Mixed/multiple ethnic groups	7.5 (9)	14.8 (18)	6.5 (2)	16.7 (8)	7.9 (7)	13.5 (10)
Black/African/Caribbean/Other black	49.2 (60)	39.2 (47)	48.4 (15)	37.5 (18)	50.6 (45)	39.2 (29)
Other	3.3 (4)	0.8 (1)	0.0 (0)	2.1 (1)	4.5 (4)	0.0 (0)
*Parent marital status*						
Married or living together	20.7 (25)	21.5 (26)	18.2 (6)	18.4 (9)	21.6 (19)	23.6 (17)
Lone parent	79.3 (96)	78.5 (95)	81.8 (27)	81.6 (40)	78.4 (69)	76.4 (55)
*Special educational needs (SEN) status*						
No provision	76.2 (93)	73.3 (88)	75.0 (24)	71.7 (33)	76.7 (69)	74.3 (55)
Receiving SEN support	23.8 (29)	26.7 (32)	25.0 (8)	28.3 (13)	23.3. (21)	25.7 (19)
*Borough*						
Enfield	19.5 (24)	17.9 (22)	15.2 (5)	18.4 (9)	21.1 (19)	17.6 (13)
Hackney	10.6 (13)	11.4 (14)	9.1 (3)	14.3 (7)	11.1 (10)	9.5 (7)
Islington	15.4 (19)	15.4 (19)	9.1 (3)	20.4 (10)	17.8 (16)	12.2 (9)
Lambeth	32.5 (40)	33.3 (41)	48.5 (16)	28.6 (14)	26.7 (24)	36.5 (27)
Waltham Forest	22.0 (27)	22.0 (27)	18.2 (6)	18.4 (9)	23.3 (21)	24.3 (18)
*Housing type*						
Owned	5.7 (7)	11.5 (14)	3.0 (1)	12.2 (6)	6.7 (6)	11.0 (8)
Other	94.3 (116)	88.5 (108)	97.0 (32)	87.8 (43)	93.3 (84)	89.0 (65)
*Housing quality*						
Good	43.1 (44)	35.8 (39)	30.8 (8)	34.9 (15)	47.4 (36)	36.4 (24)
Acceptable	29.4 (32)	44.1 (45)	34.6 (9)	41.9 (18)	30.3 (23)	40.9 (27)
Substandard	25.5 (26)	22.9 (25)	34.6 (9)	23.3 (10)	22.4 (17)	22.7 (15)
*Income (weekly, excluding housing costs)*						
≤ £150	34.2 (39)	27.9 (31)	32.3 (10)	31.1 (14)	34.9 (29)	25.8 (17)
≥ £150	65.8 (75)	72.1 (80)	67.7 (21)	68.9 (31)	65.1 (54)	74.2 (49)
*Socio-economic status [how hard it is to live on household income right now]*						
Not at all/somewhat/difficult	75.8 (91)	76.7 (92)	71.0 (22)	77.1 (37)	79.3 (69)	76.4 (55)
Very difficult/extremely difficult	22.9 (27)	23.7 (28)	29.0 (9)	22.9 (11)	20.7 (18)	23.6 (17)

The mean age of mentors was 31.4 years (range 19 to 56), and two-thirds (65.3%) were female. Just under half (49.1%) were from a minority ethnic group, and most were in full-time (85.6%) or part-time (2.7%) employment.

### Outcomes

Table 2 presents the unadjusted and adjusted mean differences at endpoint for the intervention and control arms, as well as standardized mean differences (midpoint results in Table S6). For the PSDQ Total Difficulties > threshold and TSDQ Impact > threshold, the results are presented as odds ratios (intervention: control). All randomized participants were included in analyses in the trial arms to which they were assigned (intervention *n* = 123, control *n* = 123).

**Table 2:** Intervention effect estimates at endpoint

Scale	Subscale	Intervention mean (sd)^d^	Control mean (sd)^d^	Unadjusted MD / OR	Adjusted MD/OR	*p*	Adjusted SMD
PSDQ	Total Difficulties^a^ (TD)	17.4 (6.2)	18.3 (6.6)	− 0.7	− 1.1 (− 3.2 to 1.1)	0.33	− 0.12 (− 0.38 to 0.13)
	TD > threshold	–	–	0.9	0.9 (0.4 to 1.7) ^$^	0.63	–
CHS^b^	Hope	23.1 (5.1)	22.8 (6.1)	0.2	0.8 (− 1.6 to 3.2)	0.52	0.08 (− 0.18 to 0.35)
ECBI	Intensity	137.0 (34.5)	146.4 (36.7)	− 8.4	− 8.1 (− 19.7 to 3.5)	0.17	− 0.17 (− 0.43 to 0.08)
	Problem	19.3 (10.6)	20.7 (10.4)	− 1.5	− 1.5 (− 4.5 to 1.5)	0.31	− 0.12 (− 0.36 to 0.12)
PSDQ	Conduct	3.9 (2.1)	4.2 (2.2)	− 0.3	− 0.4 (− 1.1 to 0.4)	0.31	− 0.14 (− 0.40 to 0.13)
	Emotional	3.5 (2.4)	4.0 (2.8)	− 0.4	− 0.6 (− 1.5 to 0.3)	0.21	− 0.16 (− 0.42 to 0.10)
	Hyperactivity	6.6 (2.2)	7.1 (2.0)	− 0.3	− 0.3 (− 0.9 to 0.3)	0.36	− 0.11 (− 0.35 to 0.13)
	Impact	3.9 (3.3)	4.1 (2.9)	− 0.2	− 0.6 (− 1.5 to 0.4)	0.24	− 0.16 (− 0.42 to 0.11)
	Peer	3.4 (2.2)	3.0 (2.1)	0.3	0.2 (− 0.6 to 1.0)	0.64	0.06 (− 0.20 to 0.32)
	Prosocial	6.6 (2.1)	6.5 (2.3)	− 0.1	− 0.0 (− 0.8 to 0.7)	0.98	0.00 (− 0.25 to 0.24)
SPPC^b^	Behavioral	15.0 (3.5)	15.5 (3.3)	− 0.5	− 0.4 (− 1.9 to 1.1)	0.56	− 0.08 (− 0.34 to 0.19)
	Global	18.2 (3.9)	18.3 (3.8)	− 0.1	0.1 (− 1.4 to 1.6)	0.90	0.02 (− 0.26 to 0.29)
	Scholastic	17.2 (4.0)	16.9 (3.5)	0.2	− 0.0 (− 1.6 to 1.5)	0.99	0.00 (− 0.25 to 0.24)
	Social	18.9 (3.6)	18.8 (3.8)	0.0	0.2 (− 1.5 to 1.8)	0.84	0.03 (− 0.23 to 0.28)
TSDQ	Conduct	4.1 (2.5)	4.4 (2.3)	− 0.1	− 0.3 (− 1.1 to 0.6)	0.53	− 0.08 (− 0.35 to 0.18)
	Emotional	2.4 (2.1)	3.3 (2.7)	− 0.7	− 0.6 (− 1.4 to 0.2)	0.13	− 0.20 (− 0.46 to 0.06)
	Hyperactivity	6.6 (2.5)	7.0 (2.5)	− 0.1	− 0.3 (− 1.0 to 0.6)	0.54	− 0.09 (− 0.36 to 0.19)
	Impact > threshold^c^	–	–	1.0	1.0 (0.3 to 2.7)^$^	0.92	–
	Peer	2.6 (2.1)	3.2 (2.2)	− 0.5	− 0.5 (− 1.2 to 0.3)	0.21	− 0.16 (− 0.42 to 0.09)
	Prosocial	5.5 (2.4)	5.2 (2.3)	− 0.0	− 0.0 (− 0.9 to 0.8)	0.97	− 0.01 (− 0.29 to 0.27)
	TD	15.7 (6.6)	18.0 (6.4)	− 1.3	− 1.6 (− 3.7 to 0.5)	0.14	− 0.20 (− 0.47 to 0.07)

*TD* Total Difficulties, *OR* odds ratio, *MD* mean difference, *SMD* standardized mean difference

Adjustments made for age (≥ 9 or < 9 years), gender, borough, ethnicity, SES, SEN, marital status, baseline depression, baseline value of outcome. Mean differences (intervention - control) shown except where indicated ($) as odds ratios (odds in intervention/ odds in control)

^a^Primary outcome

^b^Based on children aged 8 and over at recruitment (*n* = 185)

^c^Baseline values not available for adjustment

^s^Complete case (not imputed)

There were improvements over time on most outcomes in both intervention and control conditions (Table S7). However, there was no statistically significant difference between the intervention and control arms on the primary outcome, PSDQ Total Difficulties score at endpoint (adjusted *standardized mean difference (SMD)* = − 0.12 (95% CI − 0.38 to 0.13), *p* = 0.33). There were also no significant differences between the intervention and control arms on any secondary outcomes, including two variables in the hypothesized mechanism of change, namely child self-esteem and self-efficacy. Allowing for possible clustering owing to some children coming from the same school (cluster) made little difference to the results for any outcome (e.g., change in p from 0.33 to 0.29 on the primary outcome). Exploratory moderator analysis found no significant sub-group differences on the primary outcome for age, gender, marital status, SES, ethnicity, or PSDQ Total Difficulties score (borderline < 16 vs. abnormal ≥ 17) at baseline (Table S8). A sensitivity analysis of the primary outcome gave results with a similar interpretation for the complete case data (adjusted MD = −1.5 (95% CI −3.6 to 0.6), *p* = 0.16) compared with the imputed data (adjusted MD = − 1.1 (95% CI − 3.2 to 1.1), *p* = 0.33).

The planned CACE analysis using the primary outcome did not provide evidence that the intervention was effective (*p* = 0.50) among children who received 11 or more months of mentoring (the recommended amount) as opposed to no mentoring (MD = − 1.0, 95% CI − 4.0 to 2.0) (Table S9). Nor did the exploratory CACE analyses find that the intervention was effective on the primary outcome when delivered with stronger adherence (*p* = 0.42) or higher quality (*p* = 0.41), or when parents received extra support (*p* = 0.41), or when children attended group sessions (*p* = 0.42), or when the mentoring relationship was strong according to the child (*p* = 0.41) (Table S10).

### Missing Data

Baseline variables were largely non-missing, apart from some financial difficulty questions and 26.4% of child-reported measures (SPPC and CHS, both asked only of children aged 8 years or above at baseline). The amount of missing data increased at post-intervention (Fig. 1). The primary outcome is 33.3% missing at T3, due to loss of contact or withdrawal of participants.

### Implementation Fidelity

Of the 123 children allocated to the intervention arm, 112 (91.1%) received some mentoring (Fig. 1). Reasons for 11 children not getting any mentoring included moving away (*n* = 4), lack of interest (*n* = 3), child’s needs escalating such that provision of the intervention was deemed unsafe (*n* = 1), loss of contact (*n* = 1), and unknown (*n* = 2).

Results for implementation fidelity are presented in Table S11. There was wide variation in the time taken to match young people to a mentor (*M* = 135.4 days, *SD* = 76.6). Matching took on average just under 4 months (median = 116 days). After matching, the mentor changed in eight cases (6.5%), and the program manager changed during the intervention for 40 (35.7%) of the 112 children who received mentoring.

The mean duration of mentoring was just under 10 months, although there was considerable variation (mean (SD): 9.93 (4.23)). Mentoring sessions, designed to last between 2 and 4 h lasted about 3 h (mean (SD): 2.9 (0.6)). However, there was large variation in the dose of mentoring received, measured in terms of number of sessions (mean (SD; range): 30.5 (11.0; 2 to 51)) and total hours (mean (SD; range): 91.2 (41.5; 4 to 200)).^1^ Fewer than half (40.5%) of children received the recommended dose (≥ 35 sessions). Common reasons for missed sessions include mentor/youth illness or holiday, or parent/sibling illness, while the most common reason for youth dropping out of mentoring is change in care status (e.g., relocation, new carer unsupportive).

Just over two-fifths (43.8%) of children who received mentoring also took part in group sessions; in over half of these cases (23.2% of the total), this involved one session only (mean (SD): 1.8 (1.1)). Two-thirds (65.2%) of the parents whose children were mentored received extra support, although there was wide variation in how many hours this involved. The mean amount was just over 10 h (mean (SD): 10.6 (14.5)), but this was skewed by one outlier parent who received 106 h of support; the median amount was 6.8 h. The most common substantive themes covered in this work were parenting skills, dealing with social care and other agencies, parent physical and mental health, and financial issues (Table S12). About one in seven (15.2%) children who received mentoring took part in family groups.

The mean adherence score for one-to-one mentoring (possible range 0 to 7) was just over 4 (mean (SD) 4.1 (0.9)). All other indicators of implementation were fairly high, with limited variation, and improved over time where measures were applied on more than one occasion. These include the managers’ rating of mentoring quality (mean (SD) 25.6 (3.5); possible range 10 to 30) and supervision (7.5 (1.1); possible range 0 to 10); engagement, captured by children’s rating of their relationship with their mentor using the MYAS (37.6 (4.6) at midpoint and (38.6 (2.8) at endpoint; possible range 10 to 40); and mentors’ rating of the quality of support received from their respective Program Manager (19.2 (1.7) at midpoint and (20.1 (1.4) at endpoint; possible range 7 to 21).

### Service Use

There was reasonably substantial use of some school-based services, for example extra parent consultation with the teacher (50.0% I, 56.2% C) but no statistically significant difference between arms (Table S13). Parents reported using additional services in over half of cases (54.2% I, 53.8% C) at midpoint and/or endpoint (Table S14). Although there were differences between arms in the rates of use of different services, the only statistically significant (*p* = .047) difference concerned CAMHS, used by twice as many children in the control arm (20.9%) as in the intervention arm (10.3%). According to parents, many of these additional services were used as a result of the child’s behavior (48.8% I, 42.5% C), although there was no statistically significant difference between arms (*p* = .516).

## Discussion

Youth mentoring interventions pair participants with a caring, non-parental adult with the goal of promoting positive youth development. A recent comprehensive meta-analysis, published after the present study ended, examined all RCTs and quasi-experimental studies of intergenerational one-to-one youth mentoring programs published in the English language between 1975 and 2017 and found a statistically significant effect across all studies and all youth outcomes of 0.21 (Raposa et al. 2019). This is consistent with previous meta-analyses (cited earlier), which is notable given the stricter definition of mentoring applied and the inclusion of more recent studies of programs that incorporate evidence-based program practices rather than relying solely on practice wisdom. The authors advised that while the findings offer some support for the efficacy of youth mentoring, with even small effects potentially exerting an important influence on youth development trajectories, there is a need to remain realistic about its modest impact and seek to improve it.

Even though the Chance UK intervention incorporated features of more effective interventions identified by Dubois et al. (2011) and others (Garringer et al. 2015), and was delivered predominantly to boys (associated with more positive effects in the Dubois et al. (2011) and Raposa et al. (2019) meta-analyses), it had no statistically significant effect on the primary outcome—PSDQ Total Difficulties at endpoint—or any secondary outcomes (at any time point). The moderator analysis found no sub-group effects for age, gender, parent marital status, PSDQ Total Difficulties at baseline, SES, or ethnicity. There was also no statistically significant effect on the primary outcome for children who received a higher-fidelity version of the intervention. The results may be generalized to other ethnically diverse contexts in high-income countries in which there is mixed service provision (but not mentoring) for children with behavioral and emotional problems.

There are various possible reasons for the lack of a statistically significant effect on the outcomes, some of which are standard considerations in the context of seeking to understand null or negative effects in a trial, while others relate specifically to the mentoring literature. The first is the level of implementation fidelity, which has long been acknowledged to affect outcomes in prevention programs (Durlak and DuPre 2008). Although the quality of mentoring and engagement were generally good, adherence was rather low, suggesting that mentors might benefit from more training in using solution-focused techniques, and dose was very variable. Specifically, just under one in 10 children in the intervention arm received no mentoring and nearly two-thirds (59.5%) of those who did get mentoring received less than the recommended number of sessions. Also, although a minority of children and the majority of parents received additional support, they received relatively little when measured in terms of contact time. However, there was no effect on the primary outcome for children who received 11 or more months of mentoring (the recommended length). Nor did exploratory analyses find an effect on the primary outcome when participants (parents or children) received extra support, or when mentoring involved greater adherence or quality, or when the mentor-child relationship was stronger.

A second possible explanation for the absence of effects lies in what children in the control arm receive. It has been argued that null results might occur because services as usual—the norm for control conditions—are improving, in part informed by positive results from earlier trials (the so-called “rising tide phenomenon”—Chen et al. 2016). With the possible exception of CAMHS, there is no evidence that children in the control arm were *more* likely than intervention arm children to receive other services because they had been referred to Chance UK but had not been allocated to the mentoring arm. (Nor is there evidence that signposting by Chance UK led to children in the intervention arm accessing more services than those in the control arm.) However, future trials of mentoring interventions would do well to gather more detailed data on the nature of services as usual and the extent to which they include or resemble high-quality mentoring.

Third, it is plausible that *some* participating children had such an elevated level of need at the outset that the intervention was unable to affect it to any great degree. The intervention targeted children with behavioral and emotional difficulties, which is reasonable given that earlier meta-analyses have found stronger effects where baseline risk is higher (DuBois et al. 2011), but average baseline levels of need according to mean PSDQ and TSDQ Total Difficulties scores far exceeded the respective clinical cut-offs (i.e., located in the abnormal range). It seems plausible that some children are referred to Chance UK at a crisis point, in other words, when teachers and parents are struggling to deal with the child’s behavioral and emotional difficulties. In support of this hypothesis, DuBois et al. (2011) argued that children with deeply rooted difficulties are less likely to benefit from mentoring than those with “more intermediate levels of challenge” (p.77). In this study, an interpretation of the lack of moderator effects for PSDQ Total Difficulties at baseline is that greater behavioral and emotional difficulties prior to the mentoring did not affect outcomes. This aligns with the more recent Raposa et al. (2019) meta-analysis, which found no differences in effect sizes as a function of baseline risk (problem behaviors and receipt of free or reduced-price school lunches). However, all participants used in this comparison have elevated need (eligibility for the trial was ≥ 14 on PSDQ and ≥ 16 on TSDQ), and the interaction tests have low power, both of which could induce the null result. Future trials could usefully explore whether adding an upper threshold on an eligibility measure such as the PSDQ in order to target children with an intermediate level of need results in stronger effects.

The fourth possible explanation is that intervention content and delivery do not effectively address the risk and protective factors associated with participants’ behavioral and emotional problems. High levels of relational satisfaction, reported by participants and mentors, did not appear to translate into improved outcomes, suggesting that the nature and form of what they do together is potentially more important. This resonates with the relatively low mean score for mentors’ use of solution-focused techniques, the argument in the literature that including more systematic teaching or advocacy in mentoring interventions would enhance their outcomes (DuBois et al. 2011), and the call for stronger adherence to evidence-based practices that target specific mechanisms underlying particular youth difficulties (Raposa et al. 2019). Future trials need to ensure that interventions involve such evidence-based practices (see Garringer et al. 2015) and analyze the extent to which adherence to those practices affects outcomes.

The fact that the direction and magnitude of change in outcomes tracks quite closely across measures for both trial conditions arguably undermines any notion in this instance of the lack of intervention effects being due to other factors (design sample or methodological limitations), particularly since there is no evidence that fidelity is associated with impact.

### Strengths and Limitations

The study has significant strengths, notably the randomized design, the use of tried-and-tested measures, the strong equivalence between arms at baseline (especially on outcome variables), the collection of data from different sources (parents, teachers and children), the measurement of children’s behavior and emotional well-being in different settings (home and school), and the analysis of the relationship between different dimensions of fidelity and outcomes. The study also has limitations. First are the high and differential rates of attrition, although this did not affect the balance of the groups on baseline outcome variables. Second, endpoint outcome measures in the intervention arm were collected before mentoring finished in over two-fifths (44%) of cases owing to delays to mentoring commencing following randomization. This was because the matching process took longer than expected for many children, in part due to a lack of suitable mentors in some localities. However, the CACE analysis did not show evidence that receiving the full mentoring program provides a benefit. Third, there is little information on what exactly mentors did with children during mentoring. Chance UK follows good practice guidance in advising mentors to agree goal-orientated activities with children but the lack of prescribed activity makes it harder to monitor activity and link activities to outcomes. This is not uncommon in mentoring studies, although naturalistic observations can be used to detail activity (Keller and Pryce 2012). Fourth, there is a lack of detailed data on the content of services as usual and if they may have produced similar effects to mentoring intervention. Finally, two hypothesized mediators of intervention effect on the proximal outcomes were not measured (regulation and aspirations).

## Conclusions

There was no statistically significant effect on any outcome. Given the high level of need of children at baseline, it is possible that many participants were recruited at a point of crisis, and that this level of need in both arms naturally reduced slightly over time. Effect sizes at endpoint are small and none are statistically significant. Moreover, children in the control arm were eligible to receive services as usual, and it is reasonable to suppose that some of the regular services they received—in particular CAMHS—may have contributed to improvements in their outcomes over time. Neither is there evidence of an effect on compliers under the CACE analysis. But given the relatively serious needs of the children at recruitment, the lack of effect may be related in part to what mentors actually deliver and whether program content focuses sufficiently and efficaciously on relevant issues. Chance UK is now engaged in a process of intervention adaptation, testing, and refinement, in large part informed by the results and conclusions of this trial.

## Electronic Supplementary Material

ESM 1 (DOCX 84.5 kb)

## Footnotes

1 The data for dose refer to the dose until the end of mentoring, regardless of when endpoint data collection happened.
