# Supplementary material for: The Effectiveness of a Community-Based Mentoring Program for Children Aged 5–11 Years: Results from a Randomized Controlled Trial
Source: Prev Sci. 2020 Jul 28;22(1):100–12. doi: 10.1007/s11121-020-01132-4 (PMC7762747; doi:10.1007/s11121-020-01132-4)
Supplement: Supplementary file 1 — (DOCX 84.5 kb) [file 11121_2020_1132_MOESM1_ESM.docx]

**Supplementary online tables**

Table S1: Brief socio-demographic profiles of boroughs from which the study sample was selected

| **Borough** | **% minority ethnic backgrounds*** | **% unemployed^$^** | **Median earnings^&^** | **% with formal qualifications^#^** | **% in different types of housing^{}^** |
| --- | --- | --- | --- | --- | --- |
| Enfield | 42.3 | 3.8 | £33,110 | 4.5 no qualifications  43.4 degree/equivalent or above | 25.6 Owned outright  36.2 Being bought mortgage or loan  17.2 Rented LA (local authority) or Housing Association  21.0 Rented private landlord |
| Hackney | 43.6 | 5.9 | £35,140 | 10.8 no qualifications  49.2 degree/equivalent or above | 11.1 Owned outright  19.8 Bought mortgage or loan  45.4 Rented LA or Housing Association  23.3 Rented private landlord |
| Islington | 32.0 | 4.5 | £39,970 | 6.2 no qualifications  62.7 degree/equivalent or above | 15.4 Owned outright  23.5 Bought mortgage or loan  35.3 Rented LA or Housing Association  25.5 Rented private landlord |
| Lambeth | 41.5 | 5.9 | £38,490 | 6.2 no qualifications  65.0 degree/equivalent or above | 10.9 Owned outright  24.9 Bought mortgage or loan  29.8 Rented LA or Housing Association  34.4 Rented private landlord |
| Waltham Forest | 49.9 | 5.4 | £33,080 | 11.6 no qualifications  42.6 degree/equivalent or above | 20.6 Owned outright  29.2 Being bought mortgage or loan  19.9 Rented LA or Housing Association  30.0 Rented private landlord |

* GLA Borough Profiles: GLA datastore 2013

$ GLA Borough Profiles: Annual Population Survey, ONS, 2015

& GLA Borough Profiles: GLA estimates 2012/13

# GLA Borough Profiles: Annual Population Survey 2015

{} GLA Borough Profiles: APS 2014

Table S2: Brief socio-demographic profiles of the child population in boroughs from which the study sample was selected

| **Borough** | **% school children from minority ethnic groups*** | **% children in poverty^$^** | **Family homelessness (rate per 1,000 households)^&^** | **Children in care (rate per 10,000 population under 18)^#^** |
| --- | --- | --- | --- | --- |
| Enfield | 78.6 | 28.1 | 7.6 | 43 |
| Hackney | 84.1 | 30.2 | 6.6 | 53 |
| Islington | 73.2 | 34.5 | 2.8 | 88 |
| Lambeth | 85.7 | 27.3 | 3.1 | 73 |
| Waltham Forest | 81.9 | 24.3 | 8.9 | 42 |

* ChiMat 2015 (2016 reports)

$ ChiMat 2014

& ChiMat 2015/16

# ChiMat 2016

Table S3: Internal consistency of outcome measures

| **Measure** | **Time point** | **Cronbach’s alpha** |
| --- | --- | --- |
| Parent-report Strengths and Difficulties Questionnaire (PSDQ) | Baseline | 0.66 |
|  | Midpoint | 0.77 |
|  | Endpoint | 0.80 |
|  |  |  |
| Eyberg Child Behavior Inventory (ECBI) | Baseline | 0.95 |
|  | Midpoint | 0.96 |
|  | Endpoint | 0.95 |
|  |  |  |
| Teacher-report Strengths and Difficulties Questionnaire (TSDQ) | Baseline | 0.59 |
|  | Midpoint | 0.84 |
|  | Endpoint | 0.84 |
|  |  |  |
| Children’s Hope Scale (CHS) | Baseline | 0.71 |
|  | Midpoint | 0.76 |
|  | Endpoint | 0.77 |
|  |  |  |
| Self-Perception Profile for Children (SPPC) | Baseline | 0.86 |
|  | Midpoint | 0.87 |
|  | Endpoint | 0.85 |
|  |  |  |
| Beck Depression Inventory (BDI) | Baseline | 0.89 |
|  | Midpoint | 0.89 |
|  | Endpoint | 0.90 |

S4 Measures of the adherence and quality dimensions of implementation fidelity

| **Dimension** | **Items** | **How measure**  **is applied** | **Possible range** |
| --- | --- | --- | --- |
| Adherence | 1. Problem-free time 2. Problem-free talk 3. Finding exceptions 4. Coping questions 5. Positive feedback 6. Scaling 7. Preferred future | Program manager indicates which of the seven solution-focused tools the mentor gave specific examples of during supervision: No (=0), Yes (=1) | 0 (low) to 7 (high) |
| Quality | 1. Sessions are well planned 2. Mentor uses appropriate solution-focused components 3. Mentor focuses on child's strengths 4. Mentor supports the child to imagine and work towards their preferred future 5. Mentor supports the development and achievement of child-led SMART goals 6. Mentor uses appropriate behavior management strategies 7. Mentor models appropriate behavior 8. Mentor sets and maintains appropriate boundaries 9. Mentor engages the child in interactive tasks with a purpose 10. Mentor broadens horizons, e.g. by introducing the child to new experiences and activities | Program manager rates mentor on each item after the supervision session: Good (=3), Acceptable (=2), Improvement needed (1) | 0 (low) to 30 (high) |

Table S5: Baseline outcomes for all participants in the trial, those lost to follow-up (withdrawn or unable to contact), and those remaining in the trial to the end. Values are mean and standard deviation unless otherwise stated.

| **Baseline variable** | **All participants** | | **Participants lost to follow-up** | | **Remaining participants** | |
| --- | --- | --- | --- | --- | --- | --- |
|  | Intervention | Control | Intervention | Control | Intervention | Control |
| ***Parent-rated Strengths and Difficulties Questionnaire*** mean(sd) |  |  |  |  |  |  |
| Conduct problems | 6.0 (2.1) | 5.9 (2.3) | 6.3 (2.1) | 5.7 (2.5) | 5.9 (2.1) | 6.0 (2.2) |
| Emotional problems | 5.6 (2.4) | 5.6 (2.5) | 5.0 (2.5) | 5.6 (2.4) | 5.8 (2.4) | 5.7 (2.6) |
| Hyperactivity | 8.0 (1.7) | 8.0 (2.0) | 7.9 (1.7) | 7.8 (2.2) | 8.1 (1.8) | 8.1 (1.8) |
| Peer problems | 4.5 (2.1) | 4.3 (2.2) | 4.3 (2.1) | 4.1 (2.3) | 4.5 (2.2) | 4.4 (2.2) |
| Prosocial | 6.6 (2.2) | 6.7 (2.3) | 6.4 (2.1) | 7.1 (2.2) | 6.7 (2.3) | 6.5 (2.3) |
| Impact | 4.6 (2.4) | 4.2 (2.6) | 4.8 (2.2) | 3.6 (2.3) | 4.6 (2.4) | 4.6 (2.7) |
| Total difficulties | 24.0 (5.2) | 23.8 (5.3) | 23.5 (5.7) | 23.2 (5.6) | 24.3 (5.1) | 24.1 (5.1) |
|  |  |  |  |  |  |  |
| ***Teacher-rated Strengths and Difficulties Questionnaire*** mean(sd) |  |  |  |  |  |  |
| Conduct problems | 5.5 (2.1) | 5.8 (2.1) | 5.8 (1.9) | 5.8 (2.2) | 5.4 (2.2) | 5.8 (2.1) |
| Emotional problems | 4.6 (2.8) | 4.6 (2.7) | 4.4 (2.8) | 4.9 (2.7) | 4.6 (2.8) | 4.4 (2.8) |
| Hyperactivity | 8.2 (2.2) | 8.0 (2.0) | 8.6 (1.7) | 8.2 (1.7) | 8.0 (2.3) | 7.9 (2.2) |
| Peer problems | 4.3 (2.4) | 4.2 (2.3) | 4.1 (2.3) | 3.9 (2.2) | 4.4 (2.5) | 4.4 (2.3) |
| Prosocial | 4.2 (2.5) | 3.7 (2.3) | 3.6 (2.4) | 3.6 (2.5) | 4.4 (2.5) | 3.7 (2.1) |
| Total difficulties | 22.6 (4.8) | 22.6 (4.7) | 22.8 (5.0) | 22.8 (5.1) | 22.5 (4.7) | 22.5 (4.5) |
|  |  |  |  |  |  |  |
| ***Eyberg Child Behavior Inventory***  mean(sd) |  |  |  |  |  |  |
| Intensity | 153.7 (38.1) | 159.2 (38.4) | 157.4 (37.9) | 155.9 (42.7) | 152.3 (38.4) | 161.4 (35.5) |
| Problem | 20.7 (8.5) | 21.1 (8.4) | 19.9 (8.8) | 20.1 (8.8) | 21.1 (8.4) | 21.8 (8.1) |
|  |  |  |  |  |  |  |
| ***Self-Perception Profile for Children***  mean(sd) |  |  |  |  |  |  |
| Behavioral | 13.7 (4.1) | 14.2 (4.2) | 13.7 (5.3) | 14.0 (4.3) | 13.6 (3.6) | 14.3 (4.2) |
| Scholastic | 16.5 (4.3) | 16.0 (5.0) | 16.1 (4.5) | 16.2 (5.4) | 16.6 (4.2) | 15.9 (4.7) |
| Social | 17.0 (4.7) | 17.6 (4.8) | 17.0 (5.4) | 17.2 (5.0) | 17.0 (4.5) | 17.9 (4.6) |
| Global | 17.5 (4.4) | 18.1 (4.3) | 17.0 (5.1) | 18.6 (4.3) | 17.7 (4.1) | 17.7 (4.2) |
|  |  |  |  |  |  |  |
| ***Children’s Hope Scale***  mean(sd) | 22.4 (6.2) | 23.4 (6.2) | 23.3 (7.1) | 23.1 (6.9) | 22.1 (5.9) | 23.6 (5.7) |
|  |  |  |  |  |  |  |
| ***Beck Depression Inventory II***  mean(sd) | 9.9 (7.5) | 10.0 (7.8) | 9.1 (7.5) | 11.0 (8.5) | 10.2 (7.5) | 9.3 (7.3) |

Table S6: Analysis of intervention effects with imputed data at midpoint

| **Scale** | **Subscale** | **Intervention**  **mean (sd)** | **Control**  **mean (sd)** | **Unadjusted MD** |  | **Adjusted MD** | **p** | **Adjusted SMD** |
| --- | --- | --- | --- | --- | --- | --- | --- | --- |
| *Primary outcome* | |  |  |  |  |  |  |  |
|  | |  |  |  |  |  |  |  |
| PSDQ | TD | 18.9 (5.7) | 19.5 (6.4) | -0.5 |  | -0.9 ( -2.9 to 1.2) | 0.40 | -0.12 (-0.39 to 0.16) |
|  |  |  |  |  |  |  |  |  |
| *Secondary outcomes* | |  |  |  |  |  |  |  |
| ECBI | Intensity | 147.1 (37.3) | 146.4 (38.3) | -1.0 |  | 0.4 (-12.0 to 12.9) | 0.94 | 0.01 (-0.27 to 0.29) |
|  |  |  |  |  |  |  |  |  |
|  | Problem | 18.9 (10.6) | 20.8 (10.6) | -0.9 |  | -0.6 ( -4.0 to 2.7) | 0.70 | -0.05 (-0.32 to 0.21) |
|  |  |  |  |  |  |  |  |  |
| PSDQ | Conduct | 4.2 (2.2) | 4.5 (2.1) | -0.2 |  | -0.2 ( -0.9 to 0.6) | 0.65 | -0.06 (-0.31 to 0.20) |
|  |  |  |  |  |  |  |  |  |
|  | Emotional | 4.4 (2.5) | 4.5 (2.7) | -0.1 |  | -0.3 ( -1.1 to 0.6) | 0.56 | -0.08 (-0.35 to 0.19) |
|  |  |  |  |  |  |  |  |  |
|  | Hyperactivity | 6.5 (2.1) | 7.1 (2.3) | -0.6 |  | -0.7 ( -1.5 to 0.0) | 0.06 | -0.25 (-0.51 to 0.01) |
|  |  |  |  |  |  |  |  |  |
|  | Impact | 4.0 (2.8) | 4.3 (2.9) | 0.1 |  | -0.3 ( -1.2 to 0.7) | 0.60 | -0.07 (-0.32 to 0.19) |
|  |  |  |  |  |  |  |  |  |
|  | Peer | 3.8 (1.9) | 3.4 (2.0) | 0.4 |  | 0.3 ( -0.4 to 1.0) | 0.38 | 0.12 (-0.15 to 0.40) |
|  |  |  |  |  |  |  |  |  |
|  | Prosocial | 6.6 (2.2) | 7.1 (2.2) | -0.6 |  | -0.6 ( -1.4 to 0.1) | 0.09 | -0.24 (-0.51 to 0.04) |
|  |  |  |  |  |  |  |  |  |
|  | TD | 18.9 (5.7) | 19.5 (6.4) | -0.5 |  | -0.9 ( -2.9 to 1.2) | 0.40 | -0.12 (-0.39 to 0.16) |
|  |  |  |  |  |  |  |  |  |
| TSDQ | Conduct | 4.4 (2.4) | 4.7 (2.6) | -0.2 |  | -0.2 ( -1.1 to 0.7) | 0.65 | -0.06 (-0.33 to 0.21) |
|  |  |  |  |  |  |  |  |  |
|  | Emotional | 3.6 (2.5) | 3.8 (2.8) | 0.0 |  | 0.1 ( -0.9 to 1.0) | 0.90 | 0.02 (-0.26 to 0.30) |
|  |  |  |  |  |  |  |  |  |
|  | Hyperactivity | 6.9 (2.5) | 7.6 (2.2) | -0.6 |  | -0.5 ( -1.3 to 0.3) | 0.23 | -0.17 (-0.45 to 0.11) |
|  |  |  |  |  |  |  |  |  |
|  | Peer | 3.3 (2.1) | 3.5 (2.3) | -0.1 |  | 0.0 ( -0.7 to 0.8) | 0.93 | 0.01 (-0.25 to 0.27) |
|  |  |  |  |  |  |  |  |  |
|  | Prosocial | 5.1 (2.3) | 4.6 (2.5) | 0.4 |  | 0.3 ( -0.5 to 1.1) | 0.47 | 0.10 (-0.17 to 0.37) |
|  |  |  |  |  |  |  |  |  |
|  | TD | 18.2 (6.8) | 19.6 (6.6) | -0.9 |  | -0.6 ( -3.0 to 1.7) | 0.59 | -0.07 (-0.35 to 0.20) |
|  |  |  |  |  |  |  |  |  |

TD: total difficulties; MD: mean difference; SMD: standardised mean difference

Adjustments made for age (≥ 9 or <9), gender, borough, ethnicity, SES, SEN, marital status, baseline depression, baseline value of outcome

Mean differences (intervention - control) shown

# Based on children aged 8 and over at recruitment (n=185)

Table S7: Outcome scores at all three time points (baseline (T1), midpoint (T2) and endpoint (T3)) for intervention and control conditions. Values are mean and standard deviation unless stated otherwise.

| **Scale** | **Subscale** | **Intervention** | | | **Control** | | |
| --- | --- | --- | --- | --- | --- | --- | --- |
|  |  | T1 | T2 | T3 | T1 | T2 | T3 |
| CHS^#^ | Hope | 22.4 (6.2) | 22.68 (6.03) | 23.08 (5.10) | 23.4 (6.2) | 23.83 (5.66) | 22.82 (6.09) |
|  |  |  |  |  |  |  |  |
| ECBI | Intensity | 153.7 (38.1) | 147.10 (37.28) | 136.99 (34.45) | 159.2 (38.4) | 150.17 (38.27) | 146.35 (36.71) |
|  |  |  |  |  |  |  |  |
|  | Problem | 20.7 (8.5) | 18.86 (10.62) | 19.28 (10.64) | 21.1 (8.4) | 20.77 (10.63) | 20.66 (10.40) |
|  |  |  |  |  |  |  |  |
| PSDQ | Conduct problems | 6.0 (2.1) | 4.23 (2.23) | 4.08 (2.54) | 5.9 (2.3) | 4.46 (2.10) | 4.41 (2.32) |
|  |  |  |  |  |  |  |  |
|  | Emotional problems | 5.6 (2.4) | 4.38 (2.50) | 2.41 (2.12) | 5.6 (2.5) | 4.51 (2.74) | 3.34 (2.68) |
|  |  |  |  |  |  |  |  |
|  | Hyperactivity | 8.0 (1.7) | 6.53 (2.13) | 6.61 (2.47) | 8.0 (2.0) | 7.10 (2.33) | 6.99 (2.50) |
|  |  |  |  |  |  |  |  |
|  | Peer problems | 4.5 (2.1) | 3.76 (1.91) | 2.64 (2.13) | 4.3 (2.2) | 3.43 (1.97) | 3.22 (2.19) |
|  |  |  |  |  |  |  |  |
|  | Prosocial | 6.6 (2.2) | 6.63 (2.19) | 5.45 (2.44) | 6.7 (2.3) | 7.12 (2.16) | 5.20 (2.33) |
|  |  |  |  |  |  |  |  |
|  | Impact | 4.6 (2.4) | 3.97 (2.83) | 2.64 (1.87) | 4.2 (2.6) | 4.29 (2.91) | 3.17 (1.87) |
|  |  |  |  |  |  |  |  |
|  | Impact > threshold^$^ | 0.96 | 0.87 | 0.76 | 0.93 | 0.89 | 0.86 |
|  |  |  |  |  |  |  |  |
|  | **TD*** | 24.0 (5.2) | 18.89 (5.67) | 17.43 (6.17) | 23.8 (5.3) | 19.49 (6.40) | 18.33 (6.58) |
|  |  |  |  |  |  |  |  |
|  | TD > threshold^$^ | 0.90 | 0.69 | 0.58 | 0.89 | 0.73 | 0.61 |
|  |  |  |  |  |  |  |  |
| SPPC^#^ | Behavioural | 13.7 (4.1) | 14.21 (4.06) | 14.97 (3.46) | 14.2 (4.2) | 15.02 (3.48) | 15.45 (3.29) |
|  |  |  |  |  |  |  |  |
|  | Global | 17.5 (4.4) | 17.42 (4.03) | 18.17 (3.90) | 18.1 (4.3) | 18.25 (3.65) | 18.28 (3.79) |
|  |  |  |  |  |  |  |  |
|  | Scholastic | 16.5 (4.3) | 16.78 (4.03) | 17.15 (4.04) | 16.0 (5.0) | 15.52 (4.09) | 16.92 (3.47) |
|  |  |  |  |  |  |  |  |
|  | Social | 17.0 (4.7) | 17.96 (4.08) | 18.92 (3.61) | 17.6 (4.8) | 17.62 (4.43) | 18.82 (3.83) |
|  |  |  |  |  |  |  |  |
| TSDQ | Conduct problems | 5.5 (2.1) | 4.40 (2.40) | 4.08 (2.54) | 5.8 (2.1) | 4.73 (2.61) | 4.41 (2.32) |
|  |  |  |  |  |  |  |  |
|  | Emotional problems | 4.6 (2.8) | 3.60 (2.50) | 2.41 (2.12) | 4.6 (2.7) | 3.78 (2.77) | 3.34 (2.68) |
|  |  |  |  |  |  |  |  |
|  | Hyperactivity | 8.2 (2.2) | 6.86 (2.47) | 6.61 (2.47) | 8.0 (2.0) | 7.56 (2.23) | 6.99 (2.50) |
|  |  |  |  |  |  |  |  |
|  | Peer problems | 4.3 (2.4) | 3.30 (2.14) | 2.64 (2.13) | 4.2 (2.3) | 3.53 (2.25) | 3.22 (2.19) |
|  |  |  |  |  |  |  |  |
|  | Prosocial | 4.2 (2.5) | 5.10 (2.27) | 5.45 (2.44) | 3.7 (2.3) | 4.64 (2.47) | 5.20 (2.33) |
|  |  |  |  |  |  |  |  |
|  | Impact^&^ | - | 2.92 (1.78) | 2.64 (1.87) | - | 3.27 (1.79) | 3.17 (1.87) |
|  |  |  |  |  |  |  |  |
|  | Impact > threshold^&$^ | - | 0.89 (0.31) | 0.83 (0.38) | - | 0.92 (0.27) | 0.89 (0.32) |
|  |  |  |  |  |  |  |  |
|  | TD | 22.6 (4.8) | 18.15 (6.76) | 15.74 (6.58) | 22.6 (4.7) | 19.60 (6.58) | 17.95 (6.64) |
|  |  |  |  |  |  |  |  |
|  | TD > threshold^$^ | 0.93 | 0.68 | 0.61 | 0.97 | 0.55 | 0.45 |
|  |  |  |  |  |  |  |  |
| BDI II | BDI II | 9.9 (7.5) | 8.03 (6.43) | 5.76 (5.98) | 10.0 (7.8) | 7.46 (6.62) | - 1. 6.72) |

T2 and T3 values are complete case (i.e. not imputed) data

TD: Total difficulties

* Primary outcome

^#^ Based on children aged 8 and over at recruitment (n=185)

^&^ Baseline values not available

^$^ Proportion >=17 (for TD score) and >0 (for impact score)

Table S8: Analyses of moderators of primary outcome (PSDQ Total Difficulties at endpoint)

| **Moderator** | **Subgroup** | **Control** | | | | **Intervention** | | **Adjusted mean difference (95% CI)^#^** | | **p-value for** |
| --- | --- | --- | --- | --- | --- | --- | --- | --- | --- | --- |
|  |  | **mean (SD)^*^** | | | | **mean (SD)^*^** | |  |  | **Interaction^#^** |
| Age | < 9 years | 19.0 (6.7) | | | | 16.9 (6.7) | | -2.5 (-5.3, 0.3) | | 0.16 |
|  | ≥ 9 years | 17.5 (6.4) | | | | 17.9 (5.7) | | 0.3 (-2.7, 3.3) | |  |
|  |  |  |  | | |  |  |  |  |  |
| Gender | Male | 18.0 (6.7) | | | | 17.6 (6.1) | | 1.9 (-5.7, 9.7) | | 0.39 |
|  | Female | 20.6 (5.1) | | | | 15.9 (6.5) | | -0.7 (-3.1, 1.7) | |  |
|  |  |  | | | | | |  |  |  |
| Marital status | Married or living together | 19.8 (7.0) | | | 18.1 (5.7) | | | -1.7 (-5.6, 2.2) | | 0.72 |
|  | Lone parent | 18.2 (6.3) | | | 17.3 (6.4) | | | -0.9 (-3.3, 1.6) | |  |
|  |  |  | |  |  | |  |  |  |  |
| PSDQ TD baseline | < ‘abnormal’ threshold (<17) | 15.3 (5.6) | | | 14.7 (3.0) | | | -1.5 (-4.0, 1.0) | | 0.50 |
|  | ≥ ‘abnormal’ threshold (≥ 17) | 18.5 (6.6) | | | 17.7 (6.3) | | | 0.4 (-4.45, 5.2) | |  |
|  |  |  | |  |  | |  |  |  |  |
| SES (how hard to live on household income right now) | Not at all /somewhat/ difficult | 19.0 (6.9) | | | 17.3 (6.1) | | | -1.8 (-9.0, 5.5) | | 0.83 |
|  | Very difficult or extremely difficult | 17.2 (5.4) | | | 17.3 (6.1) | | | -0.9 (-3.1, 1.4) | |  |
|  |  |  | |  |  | |  |  |  |  |
| Ethnicity | White | 19.1(6.2) | | | 16.8 (6.7) | | | -2.1 (-5.8, 1.6) | | 0.68 |
|  | Asian/Asian British | 19.0 (5.3) | | | 20.8 (5.3) | | | 2.8 (-6.5, 12.2) | |  |
|  | Mixed / multiple ethnic groups | 20.1 (6.7) | | | 16.9 (6.7) | | | -0.8 (-7.2, 5.7) | |  |
|  | Black/African/Caribbean/any other black | 16.8 (6.1) | | | 17.6 (6.1) | | | -0.4 (-3.8, 2.9) | |  |
|  | Other | - | | | 17.3 (3.8) | | | -4.9 (-22.9, 13.2) | |  |

* complete case analysis

# imputed data analysis, with adjustments for: age, gender, borough, ethnicity, SEN, SES and marital status

Table S9: Intervention effect estimates by ITT or CACE

|  | **Control**  **mean (sd)** | **Intervention**  **Mean (sd)** | **Adjusted mean difference (95% confidence interval)^#^** | **p value** |
| --- | --- | --- | --- | --- |
| Intention to treat (ITT) | 18.3 (6.6) | 17.4 (6.2) | -1.1 (-3.2 to 1.1) | 0.33 |
| Complier Average Causal Effect (CACE) |  |  | -1.0 (-4.0 to 2.0) | 0.50 |

The CACE estimate is the estimated average effect of treatment for attending individuals if they attended fully (11 or more months of mentoring).

^#^Adjustments made for baseline variables anticipated to affect both outcome and participation: age group, depression at baseline, marital status, gender, ethnicity, SES, parent’s education (whether completed education to age 18 or beyond) and baseline PSDQ conduct.

Table S10: Further unplanned analyses of intervention effect estimates using CACE

| **Variable** | **Description of variable** | **Threshold** | **Adjusted mean difference** | **p value** |
| --- | --- | --- | --- | --- |
| Adherence | Programme manager rating of mentor’s use of solution-focused techniques (possible range 0 to 7) | ≥ 5 | -3.2 (-11.1 to 4.7) | 0.42 |
| Quality | Programme manager rating of mentor’s delivery of programme (planning and mentoring) (possible range 10 to 30, higher score is better) | ≥ 20 | -0.9 (-3.2 to 1.3) | 0.41 |
| MYAS | Child’s perception of mentoring relationship at four months (possible range 10 to 40, higher score is better) | ≥ 30 | -1.0 (-3.4 to 1.4) | 0.41 |
| Parent work | Parents received extra support (yes/no) | - | -1.4 (-4.8 to 2.0) | 0.41 |
| Child group sessions | Number of group sessions attended by children | > 0 | -1.8 (-6.1 to 2.6 ) | 0.42 |

The CACE estimate is the estimated average effect of treatment for individuals that comply. In these analyses compliance means they meet the threshold condition shown for each explanatory variable. Adjustments were made for the same variables as the planned CACE analysis (see caption to Table S9).

Table S11: Implementation fidelity

| **Fidelity measure** | **Mean (standard deviation) unless otherwise stated** |
| --- | --- |
|  |  |
| Time taken to achieve match (days) | 135.4 (76.6) |
|  |  |
| Cases where mentor changed | 6.5% (n=8) |
|  |  |
| Cases where program manager changed | 35.7% (n=40) |
|  |  |
| Duration of mentoring (months) | 9.93 (4.23) |
|  |  |
| Length of mentoring session (hours) | 2.9 (0.6) |
| < 2 hours | 6.3% |
| ≥ 4 hours | 38.7% |
|  |  |
| Duration of mentoring (sessions) | 30.5 (11.0) |
| < 12 sessions | 8.1% |
| ≥ 35 sessions | 40.5% |
|  |  |
| Duration of mentoring (hours) | 91.2 (41.5) |
| < 35 hours | 9% |
| ≥ 100 hours | 41.4% |
|  |  |
| Children received group sessions (Yes) | 43.8% |
| Number of sessions | 1.8 (1.1) |
| 1 session only | 23.2% |
|  |  |
| Parents received extra support (Yes) | 65.2% |
| Length (hours) | 10.6 (14.5) |
| < 2 hours | 20.8% |
| ≥ 7 hours | 50.0% |
|  |  |
| Children/parents attended family groups (Yes) | 15.2% |
|  |  |
| Mentoring adherence score (possible range 0 to 7) | 4.1 (0.9) |
| ≥ 5/7 | 15.2% |
|  |  |
| Rating of mentoring quality (possible range 10 to 30)* | 25.6 (3.5) |
| < 15 | 1.8% |
| ≥ 25 | 64.3% |
|  |  |
| Rating of mentor on supervision (possible range 0 to 10)* | 7.5 (1.1) |
| < 5 | 1.8% |
| ≥ 8 | 41.1% |
|  |  |
| Mentoring relationship at 4 months (possible range 10 to 40) | 37.6 (4.6) |
| ≤ 20^#^ | 2.1% |
| 40 (best possible) | 41.2% |
|  |  |
| Mentoring relationship at 9 months (possible range 10 to 40) | 38.6 (2.8) |
| ≤ 20^#^ | 0% |
| 40 (best possible) | 62.1% |
|  |  |
| Support from manager at 4 months (possible range 7 to 21)^&^ | 19.2 (1.7) |
| < 14 | 0% |
| ≥ 20 | 72.4% |
|  |  |
| Support from manager at 9 months (possible range 7-21)^&^ | 20.1 (1.4) |
| <14 | 0% |
| ≥ 20 | 74.3% |

* According to mentor supervisor

^#^ According to child being mentored

^&^ According to mentor

Table S12: Frequency of parent work themes

| **Theme** | **Frequency*** |
| --- | --- |
|  |  |
| Support, contact or administration work relating to *parenting skills* (e.g. boundaries, routines, rewards, activity-planning) and relationships with their children) | 135 |
|  |  |
| Early contact to *identify possible areas of work* with the parent/carer | 109 |
|  |  |
| Support, contact or administration work relating to parent’s *dealings with Social Care and other agencies* | 96 |
|  |  |
| Support, contact or administration work relating to *parental physical and mental health* (e.g. depression) | 94 |
|  |  |
| Support, contact or administration work relating to *financial issues* (e.g. budgeting, benefits, debt) | 92 |
|  |  |
| Support, contact or administration work relating to *parental relationship with schools* (e.g. re child’s SEN or exclusion) | 74 |
|  |  |
| Support, contact or administration work relating to *housing* issues (e.g. challenging neighbours, social housing bids) | 56 |
|  |  |
| Support, contact or administration work relating to the parent *getting back into education/training/work*; also support, contact or administration work relating to the *development of parental interests* (e.g. art, sport) | 54 |
|  |  |
| Support, contact or administration work relating to *legal issues* (e.g. immigration, domestic violence, eviction, child custody, prison visits) | 48 |
|  |  |
| *Referrals to other services* | 45 |
|  |  |
| Support, contact or administration work relating to *mentoring* (e.g. enabling sessions to happen and preparation for ending) | 36 |
|  |  |
| Other | 131 |

* Refers to the total number of times a Chance UK parent worker focused on that theme with any parent involved in the trial. One parent can work on the same theme multiple times.

Table S13: Use of school-based services in the last 6 months by children and families in the intervention and control arms (parent report at *midpoint* and *endpoint* combined)*

| **Service received** | **Intervention (%)^&^** | **Control (%)^#^** |
| --- | --- | --- |
| Extra parent consultation with head teacher | 36.9 | 31.4 |
| Extra parent consultation with class teacher | 50.0 | 56.2 |
| School nurse | 26.0 | 27.6 |
| Educational social worker | 18.0 | 24.4 |
| School doctor | 4.0 | 4.8 |
| Other school service | 39.0 | 36.5 |
| One-to-one help | 53.3 | 53.9 |
| Small group work | 57.1 | 68.5 |
| Special teaching | 23.8 | 23.6 |
| Other help at school | 19.0 | 19.1 |
| Special Educational Needs statement issued at school | 20.2 | 22.7 |
| Psychological assessment at school | 35.0 | 31.0 |
| Attended a special school | 11.7 | 17.0 |

* None of the differences between the arms are statistically significant at p<0.05

^&^ Base figure (n) ranges from 100 to 105

^#^ Base figure (n) ranges from 74 to 89

Table S14: Use of other services in the last 6 months by children and families in the intervention and control arms (parent report at *midpoint* and *endpoint* combined)

| **Service received** | **Intervention (%)^&^** | **Control (%)^#^** |
| --- | --- | --- |
| GP | 26.2 | 18.7 |
| CAMHS* | 10.3 | 20.9 |
| Hospital | 8.4 | 8.8 |
| Other health services | 14.0 | 14.3 |
| Social work | 13.1 | 9.9 |
| Educational support | 2.8 | 6.6 |
| Family or parent support | 10.3 | 7.7 |
| Art or play therapy | 2.8 | 1.1 |
| Other services | 5.6 | 8.8 |
| Any of above services used as result of child’s behavior^$^ | 48.8 | 42.5 |
| Number of services received^{}^  0  1  2  3  4  5  6  7  8 | 45.8  21.5  16.8  10.3  2.8  0.9  0  0.9  0.9 | 46.2  23.1  12.1  8.8  4.4  2.2  1.1  2.2  0 |
| Use of any additional service^^^ | 54.2 | 53.8 |

* Difference between the arms is statistically significant at p<0.05

^&^ Base figure (n) = 107 unless otherwise stated

^#^ Base figure (n) = 91 unless otherwise stated

^$^ Base figures (n) = 80 (intervention) and 73 (control)

^{}^ Base figures (n) = 107 (intervention) and 91 (control). Maximum possible number of services is 8.

^^^ Base figure (n) = 107 (intervention) and 91 (control)
